# The Anesthetist a Specialty

**Published:** 1898-10

**Authors:** 


### THE ANESTHETIST A SPECIALTY.

THE above is the title to an article by Dr. E. T. Duke in September issue of the *Maryland Medical Journal.* The article is excellent throughout and corresponds exactly to our own views on this subject. The practice of assigning the giving of the anesthetic to a young medical student, or graduate who has had no practical experience, is to our mind absolutely suicidal, and if anything unfortunate should be the result in giving the anesthetic there would be just grounds for malpractice. To our mind, in any operation the position of the anesthetist is the most important of all. Ask any physician who is going to be operated upon, and you will find that he wants to choose his own anesthetist; and yet physicians don't look at it in the same light when they are not the patient. Dr. Duke says:

"Some years spent in the study and administration of anesthetics, both in hospital and private practice, convinces me of the fact that the person performing this important duty should be specially qualified for it by study and experience. The ability of many good surgeons counts for little when the anesthetic is in the hands of an inexperienced person. This fact is well known, but so far nothing has been proposed to remedy the difficulty.

"A skilled anesthetist in every city and town, and at convenient points in the country districts, would answer the need. A theoretical knowledge of anesthetics and their administration is not sufficient. Just as a surgeon becomes skilled by constant performance of surgical operations and attention to the little details of his work, so the anesthetist by daily experience becomes perfect in his specialty, and is able to perceive intuitively the danger signals of anesthetic narcosis and employ means to avert serious results. It is this skill acquired by constant practice, coupled with study and a desire to excel, that constitutes the ideal anesthetist.

"The surgeon knowing the anesthetic is in skilled hands is relieved of any responsibility on that score and is thereby enabled to accomplish better results. Particularly is this true in private practice, where hospital facilities are not available and the surgeon is otherwise hampered in his work. In this class of work the anesthetist stands next to the surgeon in point of responsibility, and it is especially important that he should be qualified. As long as the anesthetist is made to feel his inferiority so long will he remain an inferior person. The recognition of his importance to patient and surgeon will stimulate good men to remain in or adopt this important branch of surgical work as a specialty."

---

WE note that a considerable number of the medical journals devote a large amount of space to the publication of queries and the answers thereto. They seem to pose as authorities on all subjects, and to a most incoherent question they can give a most certain and positive answer. In fact, if you will only give them the color of the patient's eyes they will write out a most extended description of the case and give the most accurate treatment in every detail. From the increase in the number of pages which these journels exhibit from time to time, it looks as if "all the fools are not yet dead," but are blissfully accepting everything that these journals tell them. It is really amusing sometimes to pick up one of these journals and notice some of the questions asked and the answers given. For instance, we find the following:

"Query 174. I have a case of chronic catarrh involving the posterior nares and pharynx; a female, aged twenty-two; family history good; menstruates regularly; health good in every other way; good appetite; a hearty, robust lady. Answer: This case is one of atrophic rhinitis (?). You have removed all obstructions from the nares, and now what remains is stimulation, in order to render the atrophied mucous membrane as active as possible. Mitchell's soluble gelatin pencils sometimes do remarkably well in such cases."

This shows exceedingly close relationship between the symptoms given and the diagnosis made.

---

THE *Times and Register*, of Philadelphia, in an editorial on the advantages offered by the New York School of Clinical Medicine to those desiring postgraduate instruction, has this unfortunate sentence: "The school knows no creed, code, or color, only requiring that the matriculant be a graduate in medicine, and is earnest in his desire to acquire knowledge." We object to such language used in reference to a school which has such excellent men in its faculty. In our last issue THE JOURNAL contained an article by Dr. Valentine, and in this issue we present to our readers a most excellent paper by Dr. Thos H. Manley, both of whom are connected with the New York School of Clinical Medicine.

The editorial, in its enthusiasm for the school, has overstepped the bounds. To say that a school is governed by "no creed" is tantamount to saying that it has no medical ethics, and when it says it is governed by "no color," then all Southern patronage must be eschewed. It is unfortunate that the editorial has made such a bold statement, as we are sure it does not represent the character of the school in question. It may be all right in Boston where the editor lives for schools to have no restriction as to the color of their students, but such schools could never be patronized from the South.

---

THERE is is not, perhaps, much in the name Smith to attract attention as a usual rule, but the name A. Lapthorn Smith, M.D., M.R.C.S., has certainly filled the pages of the medical journals for the last month. Professor Smith is one of the most brilliant Canadian gynecologists, and whenever he writes you may depend upon it as being eminently instructive. Most medical journals usually have an announcement to the effect that "no article will be allowed to appear in the Original Department which is published in any other medical journal." This rule has certainly ceased to stand in the case of Dr. Smith, for during last month we were scarcely able to pick up a single medical journal without our eyes seeing the following under "Original Articles": "Pregnancy Following Ventrofixation, with Improvements in Technique." This title has become almost as familiar as our morning paper.

---

POTT'S disease of the spine, or, in common parlance, what is known as "humpback," has in the last few years received a marked impetus in the way of treatment. Forcible reduction under an anesthetic, as first advocated and practiced by Chipault and Calôt, of Paris, has come to be an operation of recognized value. It is of course especially applicable in young children, where the vertebral structures are more pliant than at a later age. The cases of course have their limitation. Dr. Peckham, in an article in the September issue of the *Archives of Pediatrics*, has this to say in discussing the subject: "As to the cases suitable for this operation, it can easily be demonstrated, without ether, by means of extension and direct pressure, gently applied, whether or not there is any yielding. If there is, it may be inferred that the deformity is susceptible to treatment. Where there is firm anchylosis I should consider forcible correction contraindicated." This seems to be the indications and contraindications in a nutshell. It is certainly pleasant to know that there is a chance of overcoming the large number of "humpbacks" in children.

---

THE *Medical Fortnightly* for September has printed on its front cover-piece *Georgia Edition.* We have not as yet found out what these two words signify. Our first impression, upon seeing the words, was, that the contents were filled with articles written by Georgia physicians; or that it was a climatology edition, and gave the special geographic and topographic features of the Empire State of the South, showing its advantages as a winter climate; or perhaps that it gave biographical sketches of some of the noted physicians of Georgia. We have looked carefully through its pages, but nowhere can we find a reference to this State. It may perhaps have been a typographical error on the part of the printer that the word *Georgia* was printed, hence we acknowledge our ignorance in seeing any connection between the two. However, Georgians are always glad to read a good medical journal like the *Medical Fortnightly.*

---

THE Kentucky School of Medicine seems about to be swamped by internal changes. There exists, or at least did exist, two rival factions in the medical faculty, and now comes the news of a decided rupture. At a recent meeting of the Board of Regents, a majority of the Board declared vacant the chairs of Drs. Kelly and Woody, and elected Warthen, Dean, and Dr. Orendorf, Secretary, although it was claimed that Dr. Woody had previously been elected Dean, and Dr. Marvin Secretary. An injunction has been issued against Dr. Warthen to restrain him from using the office of Dean, and the decision of the court is yet to be heard from. In the meantime Dr. Jos. M. Matthews has resigned from the Kentucky School of Medicine and has been elected to the Faculty of the Hospital College of Medicine.

---

DR. COLEY, of New York, thus speaks of the operation for hernia in children: "I believe it to be a good rule never to advise operation in children until a truss has been satisfactorily tried for a reasonable time—*e.g.*, one or two years—without benefit. There are, however, important exceptions to this rule: (1) In femoral hernia operation may be at once advised, for the reason that the chances of cure by means of a truss are too slight to be considered. (2) Irreducible or adherent omentum (rare in children) and reducible hydrocele may furnish sufficient reason for early operative interference. (3) There are, furthermore, a number of cases, especially in dispensary practice, in which the rupture cannot be satisfactorily retained on account of insufficient care on the part of the parents, and such cases may with profit be subjected to immediate operation."

---

WE have received the following notice through *The Medical and Surgical Bulletin*, of Nashville:

"At the September meeting of the Medical Department of the University of Nashville, the faculty unanimously decided that all students matriculating after the present session of 1898–99 shall be required to attend four full courses of lectures before being permitted to apply for graduation."

ATTENTION is called to the meeting of the Southern Surgical and Gynecological Association, which takes place in Memphis, Tenn., on the 8th, 9th and 10th. This is one of the best associations in the United States, and its work is always of the highest order. Its Secretary, Dr. W. E. B. Davis, informs us that this promises to be one of the most successful sessions in the history of the association. Members of the medical profession are earnestly and cordially invited to attend.

---

THE State Board of Medical Examiners of this State will meet in the Senate Chamber of the State Capitol at 9 A.M., October 11, 1898. Those desiring admission to the practice of medicine in this State can then have an opportunity of standing the required examination. This journal will print the questions for that examination in the November issue.

---

YELLOW FEVER IN CUBA.—The reports, without exception, seem to show that what yellow fever there may be in and about Santiago is well in hand, and, on the whole, is diminishing. If present conditions are continued, it is safe to say that this much talked of menace to our troops has been greatly exaggerated. General Shafter reports six deaths from yellow fever in the hospital at Siboney, but none from the front. About 1,500 are sick with fever, but of these only about 150 have yellow fever. The suggestion which has been made of at once improving the drainage of Santiago should undoubtedly be carried out, whatever the general health of the inhabitants may be. Precautions of the most rigid sort are being taken to prevent the introduction of yellow fever into our southern ports. The suspicion of its appearance at Fort Monroe has not yet been confirmed, but a quarantine will be at once established should the fever actually break out. It is said that quarantine officials have refused to permit wounded soldiers to land at Old Point Comfort for fear of yellow fever.

